# Prehospital blood for the injured in conflict zones: what about civilians? - a scoping review

**DOI:** 10.1186/s13031-025-00704-x

**Published:** 2025-08-14

**Authors:** Henrik Johansson, Johan von Schreeb

**Affiliations:** 1 Department of Surgery, Helsingborgs lasarett, Helsingborg, Sweden; 2 Department of Global Public Health, Karolinska Institutet, Stockholm, Sweden

**Keywords:** Prehospital, Blood, Civilian, Conflict area, Trauma, Rural, Anesthesia, Critical care

## Abstract

**Background:**

Hemorrhage is the leading cause of preventable death in trauma patients. Prehospital transfusion (PHT) has been proposed to reduce mortality; however, its effectiveness for civilians in a military conflict zone remains uncertain due to logistical and resource constraints. While PHT is endorsed in military contexts, its routine implementation for civilian trauma care during conflicts is still debated. This study aims to explore the challenges, benefits, and limitations of PHT in civilian conflict settings based on available literature.

**Methods:**

A scoping review was conducted using PubMed, Web of Science, and Google Scholar. Peer-reviewed searches were conducted from January to February 2023, with an update in May 2024. Gray literature was reviewed in June, September, and October 2023. Studies published in English with full-text access that addressed the research question were included. Data on study design, interventions, comparisons, and outcomes were narratively synthesized.

**Results:**

Six relevant studies were identified and analyzed. Findings revealed variability in PHT practices, shaped by injury severity, transfusion protocols, and evacuation logistics. While PHT may offer benefits in settings with prolonged evacuation times, current evidence is inconsistent, limiting its routine recommendation for civilian conflict settings. Key barriers include limited resources, deviations from clinical guidelines, and challenges in blood product access and storage.

**Conclusion:**

Optimizing trauma care in civilian conflict zones requires strengthening hemorrhage control, rapid evacuation, and adaptable, context-appropriate guidelines. PHT may offer benefits in select situations, current evidence does not support its routine use. Future research should identify feasible, scalable strategies tailored to the unique logistical, ethical, and resource challenges in these settings.

## Background

Hemorrhage is the leading cause of death in trauma patients [1, 2]. Survival depends on the adequacy of hemorrhage control and resuscitation between the onset of bleeding and arrival to dedicated trauma treatment facility. The relative austerity of a treatment setting is a function of time rather than location alone, as life-saving measures must occur rapidly before hemorrhagic shock becomes irreversible [3].

In military context, far-forward resuscitation remains a present challenge in combat casualty care, highlight the need for improved field management of traumatic bleeding [3]. The U.S. Department of Defense recommends prehospital blood transfusion (PHT) for severe trauma, massive bleeding, and prolonged field care to enhance survival [4]. Data from the Global War on Terror (GWOT) indicate that PHT improves survival, with an adjusted hazard ratio of 0.26 (95% CI, 0,08 − 0,84) for the first 24 h and a 0,39 (95% CI, 0,16 − 0,92) for 30-days mortality [5, 6]. However, survival outcomes are also influenced by factors such as early treatment capability, injury type (blunt vs. penetrating), and injury body area, complicating efforts to isolate PHT’s direct effects [7]. In Ukraine, surgeons report challenges in administering PHT at the frontlines due to insufficient air support, unreliable blood supply chains, and targeted attacks on medical infrastructure during the ongoing Russian invasion [8].

In civilian trauma care, the benefits of PHT are less well established. Both prospective and retrospective studies confirm its safety, but evidence for improved survival outcomes remains limited [6, 9, 10]. Reduced mortality has been demonstrated within the first six hours of trauma, but not for 24-hours or 30-days [11]. Emerging evidence suggest PHT reduces mortality when transport times exceeds 20 min, with improvements observed both at six hours and 30 days post-injury [12, 13]. However, other studies report no clinical benefit of PHT compared to saline administration in austere environments, and transfusions themselves pose risks [3, 14].

Research on PHT in civilian trauma care faces limitations, including heterogeneous findings, incomplete data, and methodological weakness that hinder meaningful comparisons between transfused and non-transfused patients [15, 16]. These gaps complicate the development of clear evidence-based guidelines for PHT in non-military, resource-limited contexts.

The debate surrounding PHT is particularly complex in a military conflict zone, where civilian healthcare infrastructure is underfunded compared to military care (Table 1) [17, 18]. Blood products are scarce, and effective care is hampered by disrupted infrastructure, limited evacuation systems, and frequent targeting of healthcare facilities and workers [3, 8, 19–25]. Modern urban warfare results in mass civilian casualties and complex trauma patterns, demanding rapid and adaptable medical responses [26–29]. Humanitarian organizations, like Médecins Sans Frontières (MSF) and the International Committee of the Red Cross (ICRC), have deployed mobile surgical units to provide care closer to the point of injury (POI) [26–29]. Some interpretations of the Geneva Conventions suggest that frontline care may fall primarily under military responsibility, although this remains a contested and context-dependent issue [30, 31].

**Table 1:** Comparison of civilian health expenditure per capita (2021, USD) and NATO health expenditure per soldier at field hospitals (2019)

Country/Union/Organization/Alliance	USD
North America	11,818,21
European Union	4,215,43
OECD members	5,683,10
Ukraine	268,00
Fragile and conflict affected situation	82,13
NATO	≥ 20,000,00

Given these challenges, evaluating the feasibility and potential benefits of PHT for civilian trauma patients in active conflict zones is essential. A clear understanding of its limitations and logistical requirements is critical for guiding its possible implementation.

### Aim

To explore the potential benefits and drawbacks of providing prehospital blood to trauma patients within civilian conflict care as described in published articles and to discuss the role it may have in this setting.

## Methodology

This study encompasses a scoping review of both academic and gray literature, conducted in accordance with the Arksey and O’Malley framework [32], and reported following the PRISMA-ScR (Preferred Reporting Items for Systematic Reviews and Meta-Analyses extension for Scoping Reviews) guidelines [33]. Literature searches were performed in two databases, PubMed and Web of Science, along with gray literature identified through Google Scholar.

Gray literature search followed the Goldin et al. methodology for reproducibility [34]. Data were extracted from the first 100 results of the Google Scholar search. The search terms targeted key concepts such as “prehospital,” “blood,” “civilian,” and “conflict area,” which were expanded using synonyms, related medical subject headings (MeSH), a thesaurus, and free word association.

Searches for peer-reviewed articles were conducted in January and February 2023 with additional searches in May 2024. The gray literature search was performed in June, September, and October 2023. The search strategies were developed in collaboration with a medical librarian and are detailed in Table 2. Inclusion criteria mandated that studies be published in English, provide full-text access, and directly address the research topic (Table 3).

**Table 2:** Search terms used in academic databases and Gray literature searches

Source	Type of literature	Search terms
PubMed	Peer-reviewed	((“warfare and armed conflicts“[MeSH Terms] OR “warfare*“[Title/Abstract] OR “armed*“[Title/Abstract] OR “conflict*“[Title/Abstract] OR “war related injuries“[MeSH Terms] OR “war related injuries“[Title/Abstract] OR “military medicine“[MeSH Terms] OR “military medicin*“[Title/Abstract]) AND (“civil*“[Title/Abstract] OR “urban population“[MeSH Terms] OR “urban populat*“[Title/Abstract] OR “suburban population“[MeSH Terms] OR “suburban populat*“[Title/Abstract] OR “rural*“[Title/Abstract] OR “communit*“[Title/Abstract] OR “community resources“[MeSH Terms] OR “hospitals, community“[MeSH Terms] OR “community health workers“[MeSH Terms] OR “hospitals, municipal“[MeSH Terms] OR “community health workers“[MeSH Terms] OR “hospitals, municipal“[MeSH Terms] OR “hospitals, urban“[MeSH Terms] OR “urban*“[Title/Abstract] OR “public*“[Title/Abstract] OR “communal*“[Title/Abstract] OR “municipal*“[Title/Abstract]) AND (“blood“[MeSH Terms] OR “blood banks“[MeSH Terms] OR “blood volume“[MeSH Terms] OR “blood donors“[MeSH Terms] OR “blood safety“[MeSH Terms] OR “transfusion medicine“[MeSH Terms] OR “transfusion reaction“[MeSH Terms] OR “blood grouping and crossmatching“[MeSH Terms] OR “blood grouping and crossmatching“[MeSH Terms] OR “blood preservation“[MeSH Terms] OR “blood flow velocity“[MeSH Terms] OR “blood component removal“[MeSH Terms] OR “blood group incompatibility“[MeSH Terms] OR “blood transfusion“[MeSH Terms] OR “blood component transfusion“[MeSH Terms] OR “blood transfusion“[MeSH Terms] OR “plasma“[MeSH Terms] OR “blood substitutes“[MeSH Terms] OR “hemorrhage“[MeSH Terms] OR “hemorrhag*“[Title/Abstract] OR “bleed*“[Title/Abstract] OR (“blood*“[Title/Abstract] AND (“bank*“[Title/Abstract] OR “donor*“[Title/Abstract] OR “volum*“[Title/Abstract] OR “safet*“[Title/Abstract] OR “medicin*“[Title/Abstract] OR “group*“[Title/Abstract] OR “preservat*“[Title/Abstract] OR “flow*“[Title/Abstract] OR “velocit*“[Title/Abstract] OR “component*“[Title/Abstract] OR “remov*“[Title/Abstract] OR “inkompat*“[Title/Abstract] OR “transfusion*“[Title/Abstract] OR “substitite*“[Title/Abstract])))) AND (english[Filter]) + fulltext
Web of Science	Peer-reviewed	((ALL=(war) OR ALL=(warfare) OR ALL=(armed) OR ALL=(military) OR ALL=(“conflict areas”)) AND ((((((ALL=(civil)) OR ALL=(civilian)) OR ALL=(rural)) OR ALL=(urban)) OR ALL=(community)) OR ALL=(municipal))) AND ((ALL=(transfusion) OR ALL=(plasma) OR ALL=(hemorrhage)) OR ((ALL=(prehospital) AND (ALL=(blood)))
Google Scholar	Gray literature	(“prehospital” “blood” “civilian” “war” “conflict” “area”), (“prehospital” “blood” “transfusion” “civilian” “warfare” “armed” “conflict”), (“prehospital” “blood” “transfusion” “civilian” “conflict” “area”), (“prehospital” “blood” “transfusion” “civilian” “war”), (“prehospital” “blood” “transfusion” “civilian” “warfare”), (“prehospital” “blood” “civilian” “trauma” “war”), (“prehospital” “transfusion medicine” “civilian” “trauma” “war”), (“prehospital” “blood” “civilian” “trauma” “conflict” “area”), (“prehospital blood” “civilian trauma” “war”), (“prehospital blood” “civilian trauma” “conflict” “area”)

**Table 3:** Inclusion and exclusion criteria used to screen studies for the scoping review

**Critera for inclusion**
Full text
Written in English
Encompasses characteristics, concepts, elements, or components related to the subject
**Criteria for exclusion**
Abstracts

Data extraction included the title, publication year, study design, and document type, followed by extraction of information aligned with the study’s objectives. Duplicate records were manually removed. Bibliographies of relevant articles were also screened, though no additional studies were identified. Study characteristics, interventions, comparisons, and outcomes were tabulated and synthesized into a narrative summary.

## Results

**Fig. 1:**
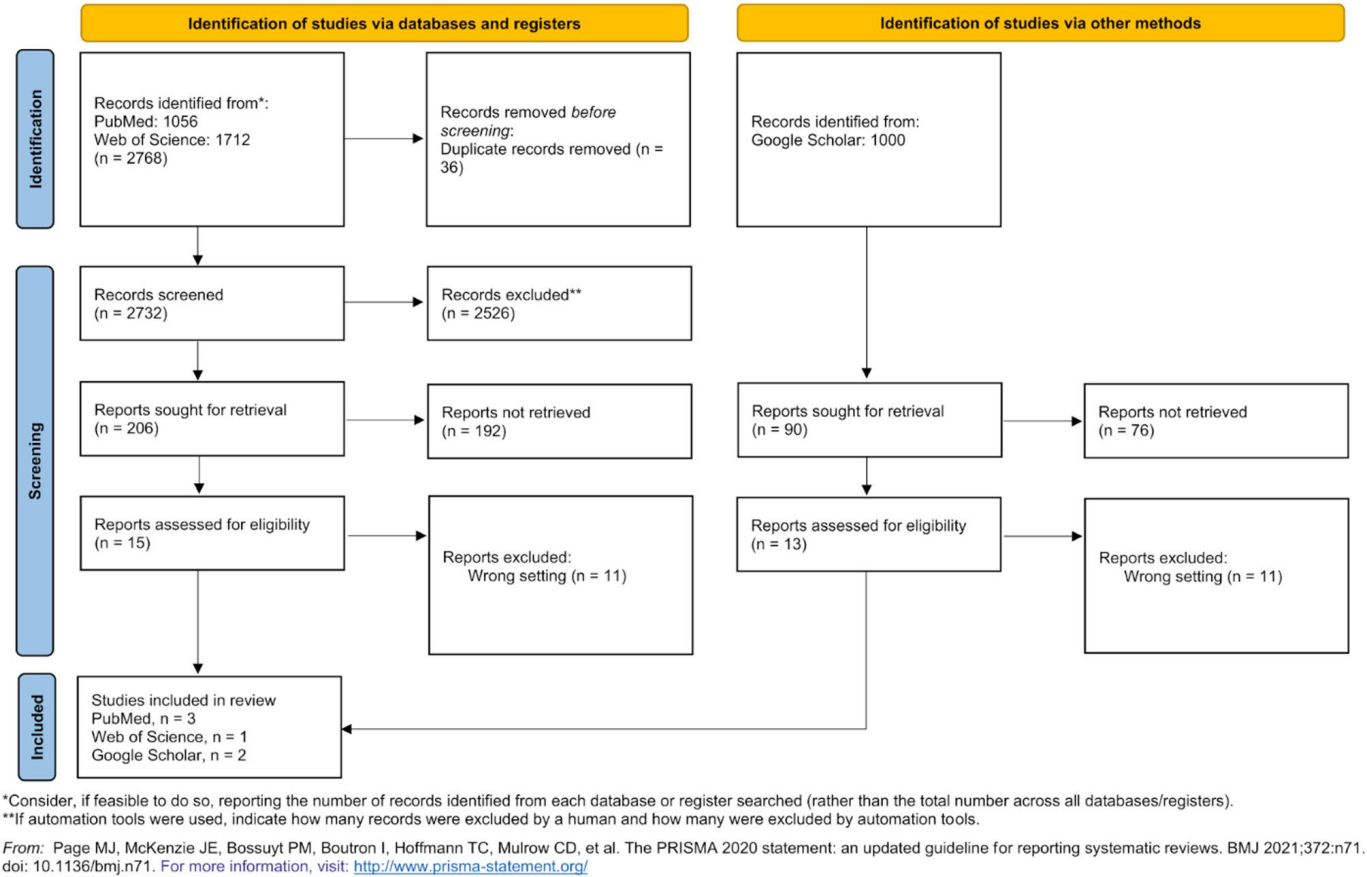
PRISMA 2020 flow diagram showing the study selection process for the scoping review

The search yielded 3,768 articles, of which 28 underwent full-text review. Ultimately, six studies met the inclusion criteria for detailed analysis (Fig. 1). These studies, conducted in conflict zones including Israel, Iraq, and Ukraine, examined the use of prehospital transfusion (PHT) in civilian trauma care (Table 4). Key challenges noted included logistical constraints, resource limitation, and risks to medical personnel in these environments.

**Table 4:** Summary of studies included in the scoping review

Year	Author	Title	Country	Population	Staff	Transport	Time period	Cohort size (civilian/soldiers)	Product type	Transfusion indication	Mean transport time	Mortality
1999	Barkna et al.	Prehospital blood transfusion in prolonged evacuation	Israel	Trauma	Military	Road/ Helicopter	30 months, 1994–1996	40 (7/33)	RBC	Hemodynamic instability after 2 l crystalloid	120 min	32,50%
2012	Murad et al.	Prehospital trauma care reduces mortality. Ten year result from a time-cohort and trauma audit study in Iraq	Iraq	Trauma	Non-grad paramedic	N/A	120 months, 1997–2006	N/A	N/A	N/A	4.4–2.3 h	17%-4%
2017	Chen et al.	Prehospital blood transfusion during aeromedical evacuation of trauma patients in Isreal: The IDF CSAR experience	Israel	Trauma	Military	Helicopter	101 months, 2003–2010	87 (48/39)	RBC	Hemodynamic instability after 2 l crystalloid	59 min	11%
2019	Benov et al.	Prehospital trauma experience of the Israel defense forces on the Syrian border 2013–2017	Isreal	Trauma	Military	N/A	57 months, 2013–2017	75 (75/0)	FDP	Change in state of consciusness, HR > 130 or BP < 90 mmHg	N/A	4,50%
2020	Garber et al.	Applying trauma systems concept to humanitarian battelfield care: a qualitative analysis of the Mosul trauma pathway	Iraq	Trauma	Not Specified	Road	10 months, 2016–2017	N/A	N/A	N/A	N/A	N/A
2023	Quinn et al.	Prehospital lessons from the war in Ukraine: Damage control resuscitation and surgery experiences from point of injury to Role 2	Ukraine	Trauma	Not Specified	N/A	13 months, 2022–2023	N/A	RBC	N/A	N/A	N/A

### Transfusion processes

Three studies from the Israel Defense Forces (IDF) reported variable outcomes with PHT. Barkana et al. (1999) conducted a 30-month (1994–1996) study involving 40 patients, including seven civilians from remote, often hostile areas. In this study, 60 units (U) of red blood cells (RBC) were administered to patients who exhibited hemodynamic instability after receiving 2 l (L) of crystalloids, with an average of 4,420 mL of crystalloid fluids administered per patient. Main mechanism of injury was penetrating (77,5%), 19 casualties of explosive devices, 9 gunshot wounds, and 3 cases of both shrapnel and bullets. The study measured injury severity using the Injury Severity Score (ISS), with a mean ISS of 18.5. The mean transport time to hospital was 120 min [35].

Chen et al. (2017) examined 87 patients over 120 months (2003–2006), during both war and peacetime, 48 of whom were civilians. The study allowed physicians to initiate RBC transfusion prior to the administration of 2 l of crystalloids if they deemed hemodynamic shock likely. Of these casualties, 98% received RBC before meeting clinical practice guidelines (CPG) criteria. Main mechanisms of injury were gunshot wounds (36%), motor vehicle accidents (28%), and explosions (24%). Lower extremities (52%) were the most injured body regions, followed by chest (45%) and abdomen (38%). The average number of injuries was 2.6 injured regions per casualty. The mean transport time was 59 min [36].

Benov et al. (2019) administered 96 U of freeze-dried plasma (FDP) to 75 of 2,785 Syrian refugees between 2013 and 2017, following IDF medical corps guidelines for hemorrhagic shock. Transfusion was performed when heart rate exceeded 130 bpm or systolic blood pressure fell below 90 mm Hg. Yet, transfusion was performed with a median heart rate of 100 bpm. In the whole group, penetrating injuries were the most prevalent, 60.4% of all injuries. Blunt injuries accounted for 16.7%. Lower extremities were the most frequently injured region (28.4%), followed by head (27.1%). Most casualties suffered injury to one body region. The average number of injuries was 1.66 per casualty. Trauma to the head was the most common injury in dead patients (61.1%). Mean transport times were not reported [37].

In Iraq, Murad et al. (2012) conducted a 10-year study (1997–2006) highlighted the challenges of using transfusions in prehospital care due to the lack of available blood products, relying instead on hypotensive resuscitation and basic life-saving measures for trauma victims. Non-graduate paramedics treated 2,788 casualties resulting from landmines, war, and traffic accidents. Most injuries were blunt (71%). The study measured injury severity using the Injury Severity Score (ISS), with a mean ISS of 6.1. 37% of the patients had serious injuries (ISS ≥ 9), and 32% were classified as major trauma victims. Extremity injuries were the most frequent (34%), while 24% of all patients had critical area injuries (head, neck, or torso). The specific amount and type of fluids administered were not provided. The mean transport time was 150 min [38].

Gaber et al. (2020) reported PHT was not routinely available during the battle of Mosul, Iraq. Fluid resuscitation was carried out during the first 10-month period of the offensive in 2016–2017 by various actors, including NGOs, UN agencies, and military forces, but detailed data on cohort size, mechanism of injury, transfusion units, or transport time were lacking [39]. Similarly, Quinn et al. (2023) provided commentary on the ongoing war in Ukraine, where PHT was recently legalized, but low-titer O whole blood was not widely available. No specific data on cohort size, mechanism of injury, transfusion units, or transport times were presented. According to the authors, using whole blood and blood products must be prioritized [40].

### Transfusion outcomes

Mortality outcomes varied across the studies. Barkana et al. reported 32,5% mortality among transfused patients, with 13 of 40 deaths, including four post-hospital admissions. Prehospital transfusion was deemed appropriate, with 21 of 31 patients receiving blood during initial in-hospital resuscitation [35]. Chen et al. observed an 11% mortality rate and concluded that PHT was safe, feasible, and potentially beneficial. To use either RBC or FDP in prehospital environments were assumed to be a necessary choice. However, the lack of a control group complicated the assessment of RBC transfusion efficacy [36].

Benov et al. reported a 4.5% mortality rate but did not specify which patients received transfusions. The use of FDP at the point of injury (POI) was considered feasible and effective with also reducing the volume of crystalloids administered. The case fatality rate (CFR) of patients treated with FDP was four (5.3%). However, the efficacy of FDP was not assessed [37]. Murad et al. achieved a significant reduction in trauma mortality, from 17 to 4%, using low-cost prehospital trauma measures like hypotensive resuscitation and compression in uncontrolled extremity bleeding patients [38].

Adverse transfusion reactions were minimal, with Barkana et al. documenting one case of a mild rash following RBC administration [35]. No other studies reported reactions [36–38]. Two studies noted substantial blood product wastage. Barkana et al. reported that less than 3.8% of RBC units were used during the study period, and Chen et al. suggested that hundreds of RBC units were likely wasted annually [35, 36].

In terms of cost, Murad et al. estimated that trauma care per patient ranged from USD 130 to 180 [38]. Gaber et al. suggested that civilian trauma response systems based on military models may have saved up to 1,800 lives, though specific data were not provided [39].

## Discussion

### Interpretation

This review highlights the variability in prehospital transfusion (PHT) practices and outcomes in civilian conflict zones. Differences in transfusion timing, adherence to clinical practice guidelines (CPGs), and injury severity contribute to inconsistent survival benefits. Reported mortality rates range widely (4.5–32.5%), suggesting that factors such as prolonged transport times and deviations from military protocols significantly affect outcomes. For instance, Barkana et al. reported only 3.8% utilization of RBC units, whereas Chen et al. documented high annual wastage, reflecting logistical inefficiencies. Transport times varied considerably (from 59 min (Chen et al.) to 150 min (Murad et al.)) directly affecting the timeliness and feasibility of PHT. These findings underscore both the complexity of implementing PHT and the broader challenge of translating military trauma strategies into fragmented civilian healthcare systems.

### Comparison to previous studies

Most PHT research originates from military settings, where care is delivered within a comprehensive trauma system spanning from the point of injury to definitive care. These systems integrate rapid evacuation, effective communication, adherence to established CPGs and real-time data collection [41]. Such coordinated approaches have been shown to improve survival through timely hemorrhage control, blood product resuscitation, and continuous monitoring [41–43].

Studies from the Israel Defense Forces (IDF) demonstrate improved outcomes when CPGs are followed rigorously, whereas civilian conflict zones often lack this level of consistency. For example, Benov et al. emphasized the utility of freeze-dried plasma (FDP) at the point of injury to reduce crystalloid administration, while Murad et al. reported reduced trauma mortality (from 17 to 4%) using low-cost approaches such as hypotensive resuscitation. 

The application of military trauma practices in civilian contexts has shown promise in select cases. Gaber et al., for instance, documented the adaptation of WHO Emergency Medical Team standards during operations in Mosul, likely saving many lives despite austere conditions [44]. These contrasting findings highlight the impracticality of directly replicating military protocols in under-resourced civilian settings. Instead, PHT effectiveness hinges on context-specific adaptations that reflect local operational realities.

### Clinical implications

Trauma care in civilian conflict zones should prioritize hemorrhage control, rapid evacuation, and context-appropriate CPGs over the routine use of PHT. Targeted PHT may offer survival benefits in prolonged transport scenarios, if implementation is supported by adequate infrastructure, trained personnel, and reliable blood storage systems. Recent conflicts, such as in Ukraine, illustrate the operational challenges of PHT in the absence of robust evacuation networks or air superiority, conditions that previously facilitated PHT in U.S. and coalition military operations during the Global War on Terror. Accordingly, efforts should focus on strengthening trauma systems, particularly supply chain logistics, personnel training, and coordinated evacuation. Rather than directly importing military models, adapting key components (such as early hemorrhage control and strategic evacuation) may yield greater benefit.

For humanitarian and non-military responders, implementing PHT requires careful consideration of logistics, personnel capacity, and blood product management. When feasible, it can complement core trauma interventions, but hemorrhage control, timely evacuation, and adaptable, context-specific guidelines should remain the primary focus. Collaboration with local health systems and adherence to ethical frameworks are essential to ensure sustainable and just implementation.

Future research should define the specific conditions under which PHT is most effective in civilian conflict settings. This includes standardized data collection, harmonized outcome measures, and cost-effectiveness analyses. Alternative resuscitation strategies (such as FDP and hypotensive resuscitation) warrant further evaluation, particularly in low-resource contexts. Investigating blood product wastage, deviations from CPGs, and logistical barriers could provide actionable insights for refining trauma protocols.

Despite updated searches through May 2024, no new studies met the inclusion criteria. This likely reflects the difficulty of conducting real-time research in active conflict zones, where security concerns, infrastructure disruption, and ethical constraints limit data collection. Innovative, field-adapted research methodologies (such as mobile data platforms) are urgently needed.

### Strengths and limitations

This review addresses a critical gap in the literature by focusing on civilian trauma care in conflict zones, synthesizing data across multiple PHT protocols to provide a more realistic view of operational challenges beyond highly controlled military environments. It also draws attention to logistical and system-level factors that are often underrepresented in prior research.

However, several limitations must be acknowledged. The included studies varied in design, methodology, and patient populations, complicating direct comparisons. Most lacked control groups, limiting the ability to assess PHT’s direct impact on survival. As a scoping review, this study aimed to map the available evidence rather than evaluate quality or infer causality, and some relevant literature may have been missed due to database and search limitations.

A large portion of the available data derives from military contexts, raising concerns about generalizability to civilian populations. Many studies involved mixed military-civilian cohorts, making it difficult to isolate civilian-specific outcomes. Retrospective designs further constrain causal interpretation, and the lack of standardized outcome measures adds to the complexity of synthesizing results.

While gray literature sources from MSF, ICRC, and WHO may contain relevant operational data, a comprehensive review of these materials was beyond the scope of this revision. These sources warrant closer examination to better capture field-level implementation challenges and practices. Ethical and operational complexities (such as the allocation of scarce resources and the blurred boundaries between humanitarian and military efforts) also remain challenging. Further investigation into these dimensions is needed to inform equitable, context-sensitive trauma care strategies.

## Conclusion

This review underscores the challenges and inconsistencies surrounding prehospital transfusion (PHT) in civilian conflict zones, where limited resources, logistical barriers, and fragmented trauma systems complicate implementation. While PHT may offer benefits in select situations (particularly during prolonged transport) current evidence does not support its routine use in these contexts. Strengthening foundational trauma care systems, including hemorrhage control, timely evacuation, and context-appropriate basic interventions, is likely to yield greater survival benefits than broad PHT deployment. To improve outcomes, protocols must be tailored to local realities rather than directly replicating military models. Future research should focus on identifying feasible, scalable strategies for trauma care delivery in conflict-affected civilian populations.

## Data Availability

No datasets were generated or analysed during the current study.
